# Evaluation of Patient Willingness to Adopt Remote Digital Monitoring for Diabetes Management

**DOI:** 10.1001/jamanetworkopen.2020.33115

**Published:** 2021-01-13

**Authors:** Theodora Oikonomidi, Philippe Ravaud, Emmanuel Cosson, Victor Montori, Viet Thi Tran

**Affiliations:** 1 Université de Paris, Centre of Research in Epidemiology and Statistics, French National Institute of Health and Medical Research, National Institute for Agricultural Research, Paris, France; 2 Clinical Epidemiology Unit, Hôtel-Dieu Hospital, Assistance Publique–Hôpitaux de Paris, Paris, France; 3 Department of Epidemiology, Mailman School of Public Health, Columbia University, New York, New York; 4 Sorbonne Paris Nord, Sorbonne Paris Cité, Assistance Publique–Hôpitaux de Paris, Avicenne Hospital, Department of Endocrinology, Research Centre in Human Nutrition–Ile de France, North Ile-de-France Integrated Obesity Centre, Bobigny, France; 5 Sorbonne Paris Nord, Centre of Research in Epidemiology and Statistics, Research Unit 1153, French National Institute of Health and Medical Research, U1125 National Institute for Agricultural Research, National Conservatory of Arts and Crafts, Bobigny, France; 6 Department of Health and Human Services, Center for Evidence and Practice Improvement of the Agency for Healthcare Research and Quality, Rockville, Maryland; 7 Knowledge and Evaluation Research Unit, Mayo Clinic, Rochester, Minnesota

## Abstract

**Importance:**

Patients will decide whether to adopt remote digital monitoring (RDM) for diabetes by weighing its health benefits against the inconvenience it may cause.

**Objective:**

To identify the minimum effectiveness patients report they require to adopt 36 different RDM scenarios.

**Design, Setting, and Participants:**

This survey study was conducted among adults with type 1 or type 2 diabetes living in 30 countries from February to July 2019.

**Exposures:**

Survey participants assessed 3 randomly selected scenarios from a total of 36. Scenarios described different combinations of digital monitoring tools (glucose, physical activity, food monitoring), duration and feedback loops (feedback in consultation vs real-time telefeedback by a health care professional or by artificial intelligence), and data handling modalities (by a public vs private company), reflecting different degrees of RDM intrusiveness in patients’ personal lives.

**Main Outcomes and Measures:**

Participants assessed the minimum effectiveness for 2 diabetes-related outcomes (reducing hypoglycemic episodes and preventing ophthalmologic complications) for which they would adopt each RDM (from much less effective to much more effective than their current monitoring).

**Results:**

Of 1577 individuals who consented to participate, 1010 (64%; 572 [57%] women, median [interquartile range] age, 51 [37-63] years, 524 [52%] with type 1 diabetes) assessed at least 1 vignette. Overall, 2860 vignette assessments were collected. In 1025 vignette assessments (36%), participants would adopt RDM only if it was much more effective at reducing hypoglycemic episodes compared with their current monitoring; in 1835 assessments (65%), participants would adopt RDM if was just as or somewhat more effective. The main factors associated with required effectiveness were food monitoring (β = 0.32; SE, 0.12; *P* = .009), real-time telefeedback by a health care professional (β = 0.49; SE, 0.15; *P* = .001), and perceived intrusiveness (β = 0.36; SE, 0.06; *P* < .001). Minimum required effectiveness varied among participants; 34 of 36 RDM scenarios (94%) were simultaneously required to be just as or less effective by at least 25% of participants and much more effective by at least 25% of participants. Results were similar for participant assessments of scenarios regarding the prevention of ophthalmologic complications.

**Conclusions and Relevance:**

The findings of this study suggest that patients require greater health benefits to adopt more intrusive RDM modalities, food monitoring, and real-time feedback by a health care professional. Patient monitoring devices should be designed to be minimally intrusive. The variability in patients’ requirements points to a need for shared decision-making.

## Key Points

**Question:**

What is the minimum effectiveness at which patients would adopt different remote digital monitoring (RDM) modalities for managing diabetes?

**Findings:**

In this survey study of 1010 adults with diabetes from 30 countries, 65% reported that they would adopt RDM even if it offered no or modest health improvements compared with their current monitoring. Participants reported that they required RDM to be more effective when they perceived it as intrusive to their lives and when it included food monitoring or real-time feedback by a health care professional.

**Meaning:**

These findings suggest that physicians should help patients select RDM modalities that align with their preferences and are unobtrusive to their lifestyle to ensure RDM adoption.

## Teaser

This survey study identifies the minimum effectiveness patients with type 1 and type 2 diabetes report they require to adopt 36 different remote digital diabetes monitoring scenarios.

## Introduction

Remote digital monitoring (RDM) is a novel care modality that is being implemented in clinical settings because of its potential benefits for improving health outcomes.^1,2,3,4,5,6,7^ RDM consists of using prescribed sensors to capture patients’ physiological and behavioral data, which can then be transmitted to their physician to complement in-person consultations or be used to offer real-time feedback provided by artificial intelligence (AI) or a clinician.^4,8,9,10,11,12,13^

As with other treatment decisions, patients decide whether to adopt RDM by weighing its benefits against its costs and inconveniences.^14^ Previous studies have identified the costs of RDM, including disruptive alerts and social stigma,^15,16,17^ which represent the intrusiveness of RDM in patients’ private lives.^18,19^ Intrusiveness can lead to nonadherence to RDM among some patients,^15,16^ but others may decide to adopt RDM despite its intrusiveness to obtain superior health benefits than those offered by the traditional care model.^20^ The magnitude of health benefits patients require to adopt RDM and the association of this requirement with the perceived intrusiveness of RDM has not been explored. To address this gap, we performed a vignette-based survey to identify the minimum effectiveness required by patients with type 1 or 2 diabetes to adopt different RDM scenarios with varying degrees of intrusiveness.

## Methods

We designed a vignette-based survey. In vignette-based surveys, participants are asked to assess a series of vignettes on a given topic. Vignettes are hypothetical scenarios in which key components (vignette factors) are varied systematically to take 1 of several prespecified options (factor levels). This allows researchers to examine participants’ assessment of both the complete vignettes and each factor level. Our vignettes described potential applications of RDM for diabetes delivered as part of patients’ usual care.

Vignette-based surveys have been widely used to examine perceptions and stated preferences.^21^ The stated preference elicited with vignettes has been validated against real-world behavior, including behaviors with high desirability bias.^22,23,24^

### Participants

A nonprobability, convenience sample of Anglophone and Francophone adults with type 1 or 2 diabetes was recruited between February and July 2019 by (1) disseminating information about the study on patient forums, Facebook groups, and diabetes-related websites; (2) email invitation to participants of the French e-cohort ComPaRe,^25^ a citizen-science project in which patients can register to participate in research; and (3) in-person recruitment in the Endocrinology Department of the Mayo Clinic (Rochester, Minnesota). By recruiting participants via different channels, we aimed to avoid a highly select sample.

Patients were directed to the study website,^26^ where they were shown a standard information sheet reporting the purpose of the study, participants’ rights and obligations, potential harms from participation, intended statistical treatment of the collected data and publication of the results, and the contact information of the researchers. After reading the information sheet, participants could select to consent to participate (this option allowed participants to access the survey questionnaire) or refuse to participate (this options led to the participant exiting the study website). The protocol was reviewed by the ethics committee of the French National Institute of Health and Medical Research, and it is available from the corresponding author. This study followed the Strengthening the Reporting of Observational Studies in Epidemiology (STROBE) reporting guideline.

### Vignette Development

First, 3 authors (T.O., P.R., V.T.T.) selected the following vignette factors and factor levels with which to develop the study vignettes, based on a review of monitoring tools available on the market and by consultation with a panel of diabetologists:

- Monitoring tools could take 1 of the following 3 levels: (1) glucose and physical activity (PA) monitoring alone; (2) glucose, PA, and occasional food monitoring, or (3) glucose, PA, and regular food monitoring.
- Duration and feedback loop could take 1 of the following 6 levels: (1) monitoring for a week before a specific consultation with feedback in consultation; (2) monitoring for a week before all consultations with feedback in consultation; (3) monitoring permanently with real-time feedback from the patient’s regular physician; (4) monitoring permanently with real-time feedback by another care professional; (5) monitoring permanently with real-time, AI-generated treatment feedback; or (6) monitoring permanently with real-time, AI-generated treatment plus lifestyle feedback.
- Data handling could take 1 of the following 2 levels: data handling by (1) a public-sector organization (eg, public hospital) or (2) a private-sector organization (eg, insurance company).

These modalities were combined in all possible ways to develop 36 complete vignette scenarios (eAppendix 1 in the Supplement).

### Data Collection

Each participant assessed 3 randomly selected vignettes by responding to 2 questions, indicating the minimum health benefit they would require to adopt the RDM as their usual care. The first question was, “How effective would this monitoring have to be in reducing the frequency of hypoglycemic episodes for you to choose it over your current way of monitoring?” The second question was, “How effective would this monitoring have to be in preventing eye complications in the future for you to choose it over your current way of monitoring?”

Participants responded using a 5-point scale (from “it could be much less effective” to “it would have to be much more effective”). We used 2 questions referring to a short-term and long-term health outcomes because people may be biased toward short-term rewards.^27^

We collected participants’ demographic characteristics and diabetes-related data as well as their perceived intrusiveness for each vignette to examine the association between intrusiveness and minimum required effectiveness (eAppendix 2 in the Supplement). Exploring the association between RDM modalities and intrusiveness was a separate objective and is reported in a different paper.^28^

### Statistical Analysis

Data were analyzed with R version 3.6.0 (R Project for Statistical Computing). Statistical significance was set at P < .05, and all tests were 2-tailed. The unit of analysis was the vignette assessment. All participants who assessed at least 1 vignette were included in the analyses.

In simple linear regression, 10 to 30 observations are required per included independent variable.^29^ Accounting for clustering (each participant was asked to evaluate 3 vignettes), we estimated that we needed 900 vignette evaluations from 300 participants.

First, we present the results for minimum required effectiveness required by participants to adopt RDM. In calculating summary statistics we grouped the following response points, which indicate that participants would adopt RDM even if it was no more effective than their current monitoring: “it could be much less effective,” “it could be somewhat less effective,” and “it would have to be just as effective.” The full data are available in eTable 1 in the Supplement. To explore how participants’ ratings varied for the same vignette, we present the number of vignettes that were simultaneously required to be just as effective as or less effective than their current monitoring by at least 25% of participants and much more effective than their current monitoring by at least 25% of participants. These thresholds were defined by the authors to present the results succinctly.

Second, we fit 2 random-intercept multivariable cumulative-link mixed models (CLMMs) to assess the association between minimum required effectiveness (as a variable with 5 levels) and the vignette factor levels, perceived vignette intrusiveness, and participant characteristics. A number identifying each participant was used as a random intercept to account for clustering. We used multiple imputation for variables with missing data. We performed a sensitivity analysis by applying the CLMM in the complete-case data set. Variable handling and model fit are described in eAppendix 3 in Supplement. Finally, we present the minimum required effectiveness by subgroups of insulin use and diabetes type.

## Results

Overall, 1010 of 1577 individuals (64%) who consented to participate assessed at least 1 vignette; 572 (57%) were women; and the median (interquartile range [IQR]) age was 51 (37-63) years (Table 1). This resulted in 2860 vignette assessments between February and July 2019 (median [IQR] assessments per vignette, 78 [77-79]) (eFigure 1 in the Supplement). Regarding clinical characteristics, 524 participants (52%) had type 1 diabetes; 723 (72%) used insulin; and 687 (68%) considered their diabetes controlled (Table 1). In terms of diabetes-related complications, 363 patients (36%) had neuropathy, 141 (14%) had kidney failure, 45 (4%) had had a heart attack, 30 (3%) had blindness, and 21 (2%) had had a stroke, with some participants reporting more than 1 complication. Participants resided in 30 countries, predominantly France (360 [36%]). Regarding questionnaire items about problem areas in diabetes, 283 of 834 participants (34%) with complete data reported that feeling burned out by the effort needed to manage diabetes posed a somewhat serious or serious problem, and 456 (55%) reported that worrying about the future and the possibility of serious complications posed a somewhat serious or serious problem.

**Table 1.** Participant Characteristics

Characteristic	Participants, No. (%) (N = 1010)^a^
Age, median (IQR), y	51 (37-63)
Gender	
Men	394 (39)
Women	572 (57)
Prefers to self-describe	44 (4)
Country of residence	
France	360 (36)
Canada	212 (21)
United States	138 (14)
Other	300 (30)
Diabetes type	
Type 1	524 (52)
Type 2	411 (41)
Other^b^	75 (7)
Uses insulin	
Insulin shots	390 (39)
Insulin pump	333 (33)
No insulin use	281 (28)
Hypoglycemic episodes experienced in past 30 d, median (IQR), No.^c^	3 (0-10)
Required assistance during a hypoglycemic episode in past 30 d^c^	40 (5)
Current use of digital monitoring tools for health or well-being purpose^d^	
Does not use them and does not intend to in the future	214 (26)
Intends to use them or uses them irregularly	142 (17)
Uses them regularly	465 (57)

^a^ Values may not add to 100% due to rounding.

^b^ Other major country contributors were the United Kingdom (108 participants), Ireland (82 participants), New Zealand (31 participants), and South Africa (18 participants).

^c^ Estimated for 834 of 1010 participants without missing data.

^d^ Estimated for 821 of 1010 participants without missing data.

### Minimum Required Effectiveness to Adopt RDM

**Figure 1.**
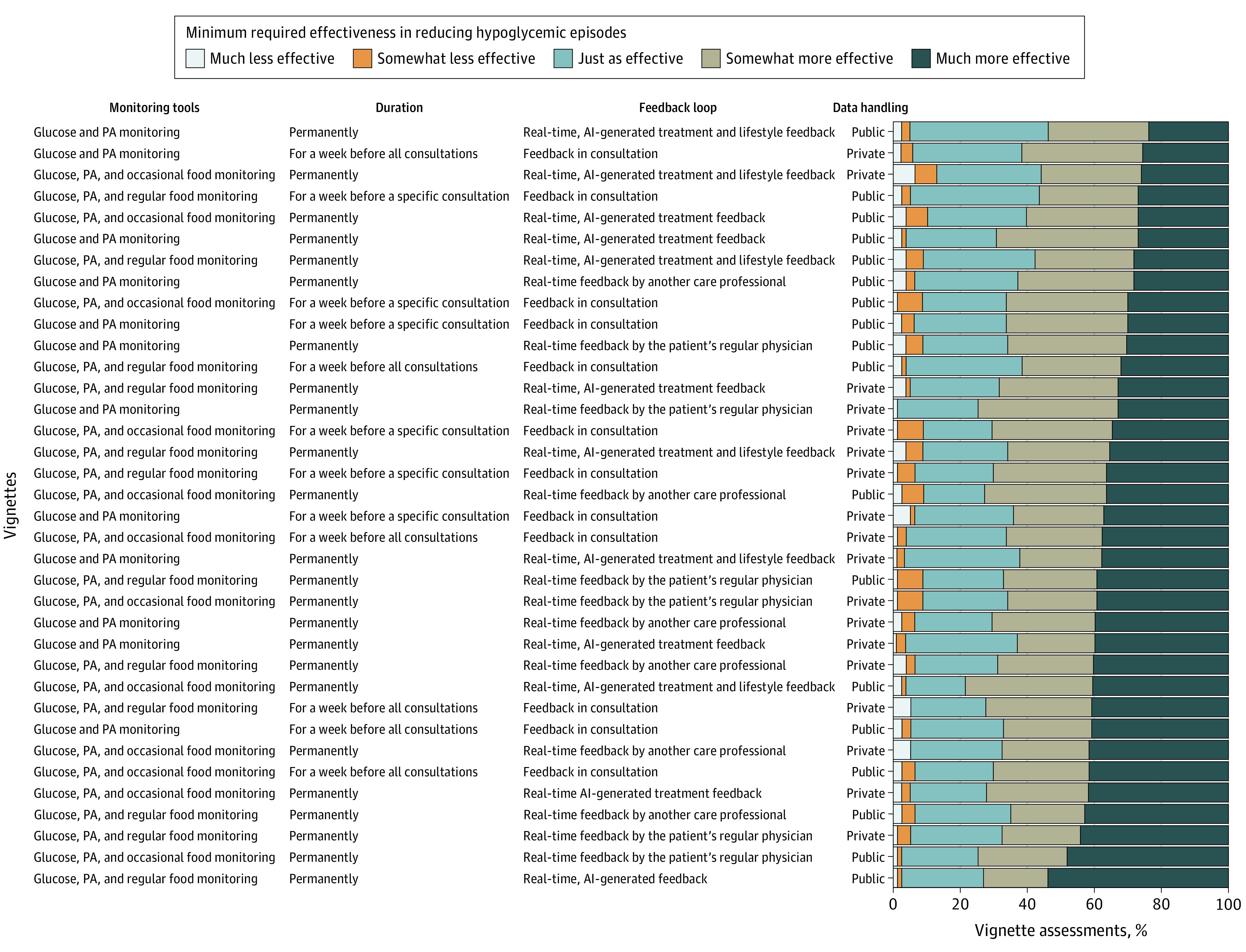
Minimum Required Effectiveness at Reducing Hypoglycemic Episodes for the Adoption of Remote Digital Monitoring in 2860 Vignette Assessments Vignettes are ranked by the proportion of assessments requiring that remote digital monitoring be much more effective. Ratings varied depending on the contents of remote digital monitoring described in different vignettes, and they varied among participants for the same vignette. AI indicates artificial intelligence; PA, physical activity.

Participants would adopt RDM in 1835 assessments (65%) if it was just as effective or less effective (959 [34%]) or somewhat more effective (876 [31%]) than their current monitoring in reducing hypoglycemic episodes, and in 1025 (36%) if it was much more effective (Figure 1; eTable 1 in the Supplement). Participants’ ratings of minimum required effectiveness varied among different vignettes. The vignette with the lowest minimum required effectiveness contained glucose and PA monitoring permanently with real-time, AI-generated treatment and lifestyle feedback and public-sector data handling. Regarding variability among participants’ views of the same RDM, 34 of 36 vignettes (94%) were simultaneously required to be just as or less effective by at least 25% of participants and much more effective by at least 25% of participants.

**Figure 2.**
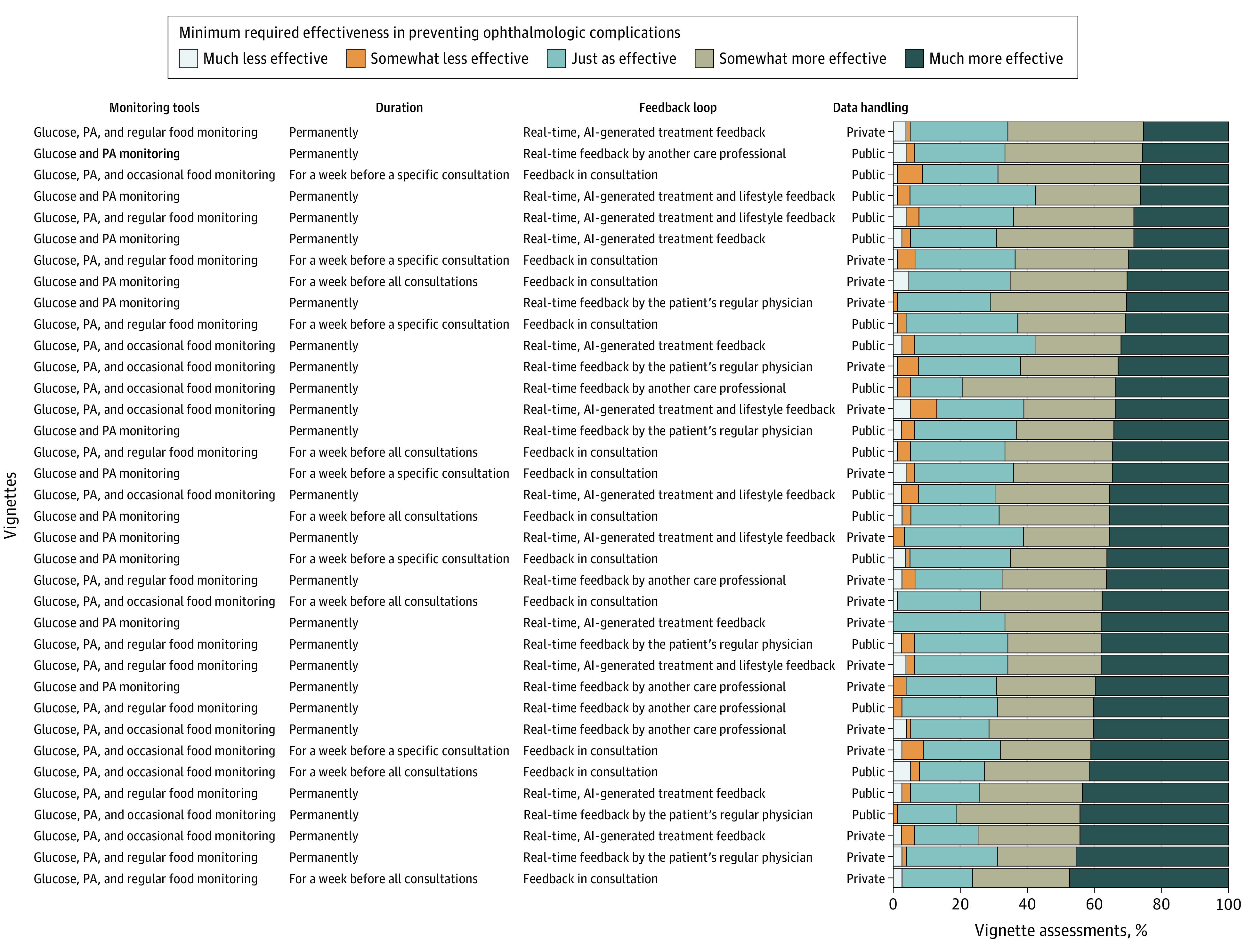
Minimum Required Effectiveness at Preventing Ophthalmologic Complications for the Adoption of Remote Digital Monitoring in 2860 Vignette Assessments Vignettes are ranked by the proportion of assessments requiring that remote digital monitoring be much more effective. Ratings varied depending on the contents of the remote digital monitoring described in different vignettes, and they varied among participants for the same vignette. AI indicates artificial intelligence; PA, physical activity.

Results were similar for preventing ophthalmologic complications (Figure 2). Participants would adopt RDM in 925 assessments (32%) if it was just as effective as or less effective than their current monitoring, in 922 (32%) if it was somewhat more effective, and in 1013 (35%) if it was much more effective. We observed variability among participants’ views of the same RDM in 33 of 36 vignettes (92%).

### Factors Associated With Minimum Required Effectiveness

Minimum required effectiveness at reducing hypoglycemic episodes was positively associated with the following vignette-level factors: RDM intrusiveness (β = 0.36; SE, 0.06; *P* < .001); glucose, PA, and occasional food monitoring (β = 0.32; SE, 0.12; *P* = .009); glucose, PA, and regular food monitoring (β = 0.28; SE, 0.12; *P* = .02); permanent monitoring with real-time feedback by the patient’s regular physician (β = 0.32; SE, 0.15; *P* = .03) or by another care professional (β = 0.49; SE, 0.15; *P* = .001); and permanent monitoring with real-time AI-generated treatment feedback (β = 0.42; SE, 0.14; *P* = .004) (Table 2). In terms of participant-level factors, minimum required effectiveness at reducing hypoglycemic episodes was associated with use of insulin shots (β = 0.81; SE, 0.27; *P* = .003) and an insulin pump (β = 1.1; SE 0.29; *P* < .001) (Akaike information criterion [AIC], 6195; *R*^2^ = 0.04).

**Table 2.** Cumulative Link Mixed Model of the Required Effectiveness Outcomes from 2860 Vignette Assessments

Factor	Reducing hypoglycemic episodes^a^			Preventing ophthalmologic complications^b^		
	β (SE)	OR (95% CI)	*P* value	β (SE)	OR (95% CI)	*P* value
Much less or somewhat less	–5.48 (0.43)	0.00 (0.00 to 0.01)	<.001	–7.12 (0.49)	0.00 (0.00 to 0.00)	<.001
Somewhat less or just as	–3.85 (0.4)	0.02 (0.01 to 0.05)	<.001	–5.31 (0.45)	0.00 (0.00 to 0.01)	<.001
Just as or somewhat more	0.16 (0.38)	1.17 (0.56 to 2.45)	.68	–0.74 (0.42)	0.48 (0.21 to 1.10)	.08
Somewhat more or much more	2.95 (0.39)	19.08 (8.96 to 40.63)	<.001	2.53 (0.43)	12.49 (5.39 to 28.96)	<.001
Vignette-level factors						
Monitoring tools						
Glucose and PA	NA	1 [Reference]	NA	NA	1 [Reference]	NA
Glucose, PA, and regular food monitoring	0.28 (0.12)	1.33 (1.05 to 1.69)	.02	0.24 (0.13)	1.28 (0.99 to 1.64)	.06
Glucose, PA, and occasional food monitoring	0.32 (0.12)	1.37 (1.08 to 1.74)	.009	0.27 (0.13)	1.31 (1.02-1.68)	.03
Duration an d feedback loop						
1 Week, with feedback given in consultation	NA	1 [Reference]	NA	NA	1 [Reference]	NA
Permanently, with real-time feedback by the patient’s regular physician	0.32 (0.15)	1.38 (1.03 to 1.84)	.03	0.23 (0.14)	1.26 (0.95 to 1.66)	.11
Permanently, with real-time feedback by another care professional	0.49 (0.15)	1.64 (1.21 to 2.20)	.001	0.48 (0.14)	1.62 (1.22 to 2.15)	.001
Permanently, with real-time, artificial intelligence-generated treatment feedback^c^	0.42 (0.14)	1.52 (1.15 to 2.02)	.004	NA	NA	NA
Permanently, with real-time, artificial intelligence-generated treatment and lifestyle feedback^c^	0.17 (0.15)	1.18 (0.89 to 1.58)	.25	NA	NA	NA
Intrusiveness rating	0.36 (0.06)	1.44 (1.29 to 1.60)	<.001	0.36 (0.06)	1.44 (1.28 to 1.62)	<.001
Participant characteristics						
Use of monitoring tools						
Does not use them and does not intend to	NA	1 [Reference]	NA	NA	1 [Reference]	NA
Intends to use them for health or well-being purposes or uses them irregularly	–0.4 (0.25)	0.67 (0.41 to 1.08)	.10	– 0.46 (0.27)	0.63 (0.37 to 1.08)	.09
Feeling burned out by the constant effort needed to manage diabetes^c^	0.2 (0.11)	1.22 (0.98 to 1.51)	.07	NA	NA	NA
Worrying about the future and the possibility of serious complications	0.17 (0.12)	1.18 (0.94 to 1.49)	.15	0.36 (0.11)	1.43 (1.15 to 1.78)	.001
Insulin use						
None	NA	1 [Reference]	NA	NA	1 [Reference]	NA
Insulin shots	0.81 (0.27)	2.24 (1.31 to 3.83)	.003	0.67 (0.31)	1.96 (1.07 to 3.59)	.03
Insulin pump	1.1 (0.29)	2.99 (1.69 to 5.28)	<.001	0.68 (0.32)	1.97 (1.04 to 3.72)	.04
Country of residence						
France	NA	1 [Reference]	NA	NA	1 [Reference]	NA
Countries other than France, United States, and Canada^d^	–0.33 (0.35)	0.50 (0.29 to 0.85)	.01	–1.29 (0.31)	0.27 (0.15 to 0.51)	<.001
United States	–0.21 (0.3)	0.72 (0.36 to 1.42)	.34	–1.17 (0.39)	0.31 (0.14 to 0.67)	.003
Canada	–0.69 (0.27)	0.81 (0.45 to 1.47)	.49	–0.41 (0.35)	0.67 (0.34 to 1.32)	.24
Gender^c^^,^^e^						
Woman	NA	1 [Reference]	NA	NA	1 [Reference]	NA
Prefers to self-describe	–0.25 (0.53)	0.78 (0.28 to 2.20)	.64	NA	NA	NA

Abbreviations: NA, not applicable; OR, odds ratio; PA, physical activity; SE, standard error.

^a^ Akaike information criterion, 6195; pseudo-*R*^2^ = 0.04 (estimated for the model vs the null using Nagelkerke method). *P* values estimated by Satterthwaite 2-sample *t* test for degrees of freedom.

^b^ Akaike information criterion, 5863; pseudo-*R*^2^ = 0.04 (estimated for the model vs the null using Nagelkerke method).

^c^ This variable was not included in the final model for minimum required effectiveness in preventing ophthalmologic complications.

^d^ Other major country contributors were the United Kingdom, Ireland, New Zealand, and South Africa.

^e^ The category men was removed in stepwise model fitting.

Minimum required effectiveness at preventing ophthalmologic complications was positively associated with the following vignette-level factors: RDM intrusiveness (β = 0.36; SE, 0.06; *P* < .001); glucose, PA, and occasional food monitoring (β = 0.27; SE, 0.13; *P* = .03); and permanent monitoring with real-time feedback by a care professional besides the participant’s regular physician (β = 0.48; SE, 0.14; *P* = .001) (Table 2). In terms of participant-level factors, it was associated positively with worry about future complications (β = 0.36; SE, 0.11; *P* = .001), use of insulin shots (β = 0.67; SE 0.31; *P* = .03), and use of an insulin pump (β = 0.68; SE, 0.32; *P* = .04) and negatively with residing in the United States (β = −1.17; SE, 0.39; *P* = .003) (AIC, 5863; *R*^2^ = 0.04).

The sensitivity analysis in the complete-case data set is presented in eTable 2 in the Supplement. For the minimum required efficacy in reducing hypoglycemic episodes, the model identified the same factors as in the imputed data set, with the exception of permanent monitoring with real-time feedback by the patient’s regular physician. For the minimum required efficacy in preventing ophthalmologic complications, the model identified the same factors as in the imputed data set, with the exception of glucose, PA, and regular food monitoring and the use of insulin shots.

### Minimum Required Effectiveness by Insulin Use and Diabetes Type Subgroups

Participants who did not use insulin required, overall, lower minimum effectiveness to adopt RDM compared with participants who used insulin shots or an insulin pump (did not use insulin and required RDM to be much more effective, 244 of 824 vignette assessments [30%]; used insulin shots, 387 of 1089 [36%]; used an insulin pump, 390 of 935 [42%]) (eFigure 2 in the Supplement). Similar differences were observed between subgroups regarding preventing ophthalmologic complications (participants who did not use insulin required RDM to be much more effective in 266 assessments [32%] compared with 379 [35%] and 362 [39%] for those who used insulin shots and an insulin pump, respectively) (eFigure 3 in the Supplement). Participants’ views of the same RDM varied for least half of the 36 vignettes in all participant subgroups.

We found little difference between participants with type 1 and type 2 diabetes in minimum required effectiveness for both outcomes (eFigure 4 and eFigure 5 in the Supplement). Participants’ views of the same RDM varied for at least 23 of the 36 vignettes (64%) in all participant subgroups.

## Discussion

This large, international study found that many participants would be willing to adopt RDM in their regular diabetes care if it were no more or somewhat more effective in improving health outcomes. However, one-third required that RDM be much more effective than their current diabetes monitoring to adopt it. The minimum effectiveness required to adopt RDM was significantly associated with RDM intrusiveness, and it varied widely among individuals for the same RDM scenario.

These findings are encouraging for the future use of RDM. Two-thirds of participants would adopt RDM if it were somewhat more effective than their current care at improving health outcomes, which may be feasible with existing technologies,^2,7,20,30,31^ although there is conflicting evidence.^20,30,32,33,34^ Half of these participants would adopt RDM even if it were no more effective than their current care, potentially motivated by other benefits of RDM (eg, reassurance). Additionally, we found that effectiveness requirements for the same RDM differed substantially among individuals, possibly due to differences in psychosocial characteristics.

RDM that was perceived as more intrusive by participants and RDM that included occasional food monitoring and real-time feedback by another care professional was required to be more effective to be adopted. Thus, patients consider intrusiveness a cost, and they may adapt their requirements for RDM benefits accordingly. Food monitoring and real-time feedback may be considered undesirable because patients worry that they may be judged for their diabetes self-management. Insulin use was significantly associated with both outcomes, possibly because diabetes management is more burdensome for those who use insulin than for those who do not, which could be taken into account in their decision to adopt a burdensome RDM regime. The model for preventing ophthalmologic complications additionally identified worry about future complications and residence in the United States as significant factors. This finding may be confounded by the fact that participants from the United States were younger and more frequently had type 1 diabetes. Worry about future complications corresponds to ophthalmologic complications being a long-term outcome.

A comparison with previous studies is difficult because the benefits patients require to adopt RDM have not been studied. Some randomized clinical trials have reported low adherence to RDM interventions,^15,33,35^ whereas others have reported high acceptability.^31,36,37,38^

RDM holds promise for patients and physicians. First, technological developments could lead to less intrusive monitoring, thereby reducing the magnitude of health benefits required to adopt RDM. Second, patients who require substantial benefits to adopt RDM could benefit from interventions designed to reduce barriers to RDM adoption.

When implementing digital diabetes care, physicians should be aware of the variability in patients’ requirements of RDM. Our results show that acceptability of RDM is contingent on how it affects health outcomes that are important to patients and how patients perceive its psychological costs. Therefore, physicians should first discuss the expected efficacy of RDM with patients and codefine treatment goals. Physicians may then use shared decision-making aids, similar to the vignettes used in this study, to help patients select the monitoring modalities that align with the benefits motivating them to adopt RDM and that carry the smallest psychological costs.

Our study focused on RDM adoption. Future studies should examine the association of RDM modalities, intrusiveness, and perceived effectiveness with sustained adherence to RDM. Adherence to digital diabetes technologies tends to decline over time,^39,40,41^ and it may be affected by intrusiveness.^18^ Additionally, this study focused on RDM as part of patients’ follow-up in the context of health care institutions. Patients’ views of using these technologies for self-management without physician involvement may differ. Future studies could also investigate issues around data handling. The balance between privacy protections, trust in private-sector organizations, and increased usability of digital health platforms (eg, by facilitating interoperability) should be examined. Finally, this is a preliminary overview of patients’ perceptions of RDM. Experimental studies are needed to test patients’ adoption of RDM in a real-world clinical context.

### Strengths and Limitations

This study has several strengths. First, this was a large, international study with participants from different countries and health care systems within the Western world. Second, the large sample allowed for precise outcome estimates. Third, the RDM vignettes represent existing sensors and applications. Fourth, the use of vignettes, a methodologically robust tool, allowed us to compare 36 diverse RDM scenarios.

Our study also has some limitations. First, our convenience sample is not representative of the 425 million people with diabetes worldwide. However, a representative sample of this size would have led to small subgroups of populations for whom RDM is highly relevant. Because our aim was to identify characteristics that may affect patients’ views of RDM, we recruited a diverse sample in terms of the characteristics whose association with the outcomes we aimed to assess. Second, our sample does not represent the patients who are currently more likely to be offered RDM in clinical settings but rather presents the views of patients with diabetes in general. We decided to explore the perceptions of these patients, for whom use of RDM is likely to be expanded in the future. Third, some characteristics expected to be associated with RDM adoption (eg, frequency of hypoglycemic episodes, current use of digital monitoring tools) may not have been strongly associated with RDM adoption because of limited variability. Results could differ in other populations. Fourth, many study participants were familiar with the use of digital health tools. Therefore, acceptability rates in the overall population of patients with diabetes may be lower than those suggested by our findings. Fifth, the proportional odds assumption did not hold for a subset of factors in the CLMM. Even when the assumption is not met, the CLMM provides a reliable unified average odds for the association between factors and the outcome variable.^42^ However, the association of factors to specific levels of the outcome variable may not be reliable.

## Conclusions

There is potential for large-scale implementation of RDM in diabetes care. The findings of this study suggest that RDM modalities that are seen as intrusive by patients may lead to greater requirements of health benefits to offset the psychological costs of RDM adoption. The variability in patients’ preferences should be considered in the design of minimally disruptive digital health tools as well as by physicians prescribing RDM.

## Notes

Supplement. **eAppendix 1. **Vignette Example **eAppendix 2. **Data Collection and Piloting **eAppendix 3. **Model Description **eTable 1. **Distribution of Minimum Required Effectiveness Ratings per Vignette **eTable 2. **Cumulative Link Mixed Model in the Complete-Case Data Set **eFigure 1. **Study Flowchart **eFigure 2. **Subgroup Analysis by Insulin Use Subgroup for Reducing Hypoglycemic Episodes **eFigure 3. **Subgroup Analysis by Insulin Use Subgroup for Preventing Ophthalmologic Complications **eFigure 4. **Subgroup Analysis by Diabetes Type Subgroup for Reducing Hypoglycemic Episodes **eFigure 5. **Subgroup Analysis by Diabetes Type Subgroup for Preventing Ophthalmologic Complications
