# Supplementary material for: Evaluation of Patient Willingness to Adopt Remote Digital Monitoring for Diabetes Management
Source: JAMA Netw Open. 2021 Jan 13;4(1):e2033115. doi: 10.1001/jamanetworkopen.2020.33115 (PMC7807289; doi:10.1001/jamanetworkopen.2020.33115)
Supplement: Supplement. — eAppendix 1. Vignette Example eAppendix 2. Data Collection and Piloting eAppendix 3. Model Description eTable 1. Distribution of Minimum Required Effectiveness Ratings per Vignette eTable 2. Cumulative Link Mixed Model in the Complete-Case Data Set eFigure 1. Study Flowchart eFigure 2. Subgroup Analysis by Insulin Use Subgroup for Reducing Hypoglycemic Episodes eFigure 3. Subgroup Analysis by Insulin Use Subgroup for Preventing Ophthalmologic Complications eFigure 4. Subgroup Analysis by Diabetes Type Subgroup for Reducing Hypoglycemic Episodes eFigure 5. Subgroup Analysis by Diabetes Type Subgroup for Preventing Ophthalmologic Complications [file jamanetwopen-e2033115-s001.pdf]

Supplemental Online Content

Oikonomidi T, Ravaud P, Cosson E, Montori V, Tran VT. Evaluation of patient willingness to adopt remote digital monitoring for diabetes management. *JAMA Netw Open*. 2021;4(1):e2033115. doi:10.1001/jamanetworkopen.2020.33115

eAppendix 1. Vignette Example

eAppendix 2. Data Collection and Piloting

eAppendix 3. Model Description

eTable 1. Distribution of Minimum Required Effectiveness Ratings per Vignette

eTable 2. Cumulative Link Mixed Model in the Complete-Case Data Set

eFigure 1. Study Flowchart

eFigure 2. Subgroup Analysis by Insulin Use Subgroup for Reducing Hypoglycemic Episodes

eFigure 3. Subgroup Analysis by Insulin Use Subgroup for Preventing Ophthalmologic Complications

eFigure 4. Subgroup Analysis by Diabetes Type Subgroup for Reducing Hypoglycemic Episodes

eFigure 5. Subgroup Analysis by Diabetes Type Subgroup for Preventing Ophthalmologic Complications

This supplemental material has been provided by the authors to give readers additional information about their work.

eAppendix 1. Vignette Example

Imagine that your doctor prescribes that you use the diabetes monitoring below, at no additional financial cost to you.

Scenario 1/3:

Digital tools:

- A glucose sensor and an app to monitor your physical activity.
- An app to monitor your food intake. You will have to take pictures of only the meals, snacks or drinks that are unusual to what you ordinarily consume.

Monitoring duration:

- This will be your regular monitoring from now on.

Adapting your treatment:

- Your data will be used to automatically adapt your treatment. This information will appear on your smartphone in real time.
- No regular visits will be required to follow-up on your diabetes, but you will be able to make an appointment with your doctor if you wish to.
- Your doctor will not receive any real-time notifications.

Data handling:

- Your data will be handled by a private organization (an insurance, a pharmaceutical or an informatics company).

1. How intrusive would this diabetes monitoring be to your daily life? *

☐	☐	☐	☐	☐
Not at all	A little	Moderately	Very	Extremely

2. How reassured would this monitoring make you feel? *

☐	☐	☐	☐	☐
Not at all	A little	Moderately	Very	Extremely

© 2021 Oikonomidi T et al. *JAMA Network Open.*

3. How effective would this monitoring have to be at reducing the frequency of hypoglycaemic episodes (low glucose levels) for you to choose it over your current way of monitoring? *

☐	☐	☐	☐	☐
It could be much less effective	It could be somewhat less effective	It would have to be just as effective	It would have to be somewhat more effective	It would have to be much more effective

4. How effective would this monitoring have to be at preventing eye complications in the future for you to choose it over your current way of monitoring? *

☐	☐	☐	☐	☐
It could be much less effective	It could be somewhat less effective	It would have to be just as effective	It would have to be somewhat more effective	It would have to be much more effective

* Required responses

© 2021 Oikonomidi T et al. *JAMA Network Open.*

eAppendix 2. Data Collection and Piloting

I. Collected data

Participants' perceptions of intrusiveness for each vignette were collected using the question "How intrusive would this monitoring be to your daily life?" (response range "Not at all" to "Extremely"). We collected the following demographic and diabetes-related data: age, sex, education, country, diabetes type, insulin use, number of hypoglycemic episodes in the past month, 3 items from the Problem Areas In Diabetes [PAID] scale on burnout, guilt, and worry about complications, intrusiveness rating of participants' current monitoring, and participants' current use of digital monitoring tools.

II. Piloting

The survey was drafted in English and translated to French by a bilingual author (TO). The translation was compared to the original version by two bilingual French speakers. The survey was pilot-tested with 3 participants (2 women with type 1 diabetes, 1 man with type 2 diabetes).

© 2021 Oikonomidi T et al. *JAMA Network Open.*

eAppendix 3. Model Description

I. Multiple imputation

Data were missing in 418 observations (i.e., vignette assessments) for the following pre-specified candidate predictor variables of the LMM model: number of hypoglycemic episodes in the past 30 days, Problem Areas In Diabetes (PAID) questionnaire items on burnout and worry, and current use of monitoring tools. Because all of these questions were presented in the final page of the survey, we assumed data to be missing not at random (e.g., due to participant fatigue). Data were missing from the variable insulin use for 4 observations owing to a javascript error in the survey website.

We present the characteristics of participants with complete and incomplete data below. We used the mice R package to impute missing data with 30 iterations. The number of hypoglycemic episodes in the past 30 days, and the two PAID questionnaire items were imputed by using predictive means matching. Current use of monitoring tools was imputed by using polytomous regression.

Multiple imputation drew information from the following variables (which were present for all participants): age, sex, country of residence, diabetes type, whether the participant considered their diabetes to be well controlled or not, insulin use, and outcome data (intrusiveness and minimum required efficacy scores). A participant identifier variable was used as the grouping variable to indicate the clustered structure of our data (i.e., several vignettes assessed by the same participant).

Table: Characteristics of participants with complete and incomplete data

**Characteristics**	**Complete data (n=814)**	**Incomplete data (n=196)**
Age, median (IQR)	51 [37, 63]	52 [37, 64]
Gender, No. (%)		
Male	311 (38)	83 (42)
Female	471 (58)	101 (52)

© 2021 Oikonomidi T et al. *JAMA Network Open.*

Prefers to self-describe	32 (4)	12 (6)
Country of residence, No. (%)		
France	301 (37)	61 (32)
Canada	171 (21)	40 (21)
United States	110 (14)	28 (15)
United Kingdom	86 (11)	22 (11)
Ireland	60 (7)	22 (11)
Other	84 (10)	20 (10)
Post-secondary education	584 (71)	143 (73)
Diabetes type, No. (%)		
Type 1	423 (52)	101 (52)
Type 2	340 (42)	71 (36)
Other	51 (6)	24 (12)
Considers diabetes well-controlled	559 (69)	128 (65)
Uses insulin, No. (%) [a]		
Yes, shots	303 (37)	86 (45)
Yes, pump	272 (33)	59 (31)
No	239 (29)	47 (24)

[a] missing in n=4 of participants with incomplete data

© 2021 Oikonomidi T et al. *JAMA Network Open.*

II. Distribution of the dependent variables

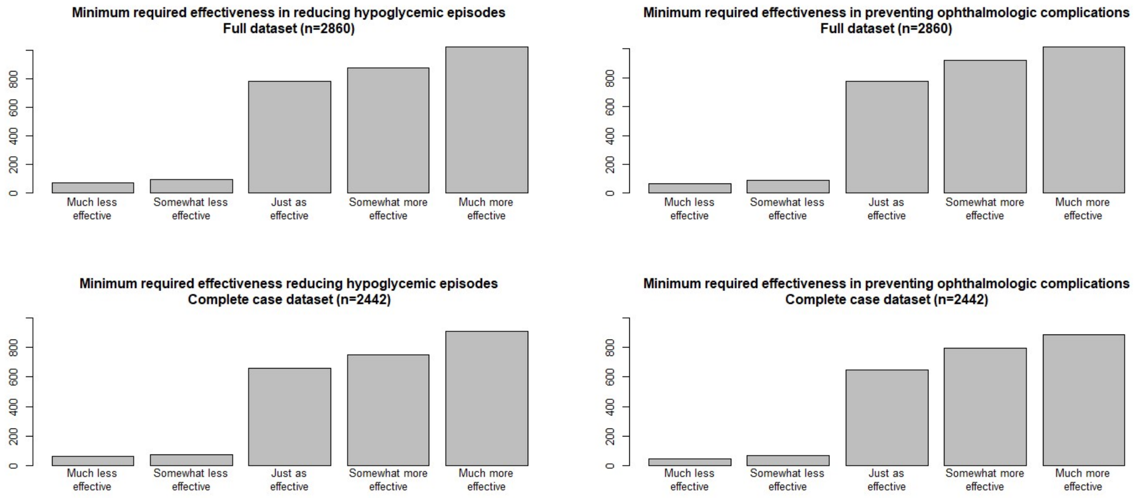

III. Variables entered in the model

All independent variables for both models were selected on the basis of previous publications and clinical experience by the authors.[1–3]

The following independent variables were entered in both theory-driven models: vignette factor 1 (monitoring tools, categorical variable with 3 categories), vignette factor 2 (duration/feedback loop, categorical with 6 categories), vignette factor 3 (data handling, binary), intrusiveness (continuous), age (continuous), insulin use (categorical with 3 categories: no insulin, shots, pump), country (categorical with 4 categories: France, United States, Canada, other), number of hypoglycemic episodes in the last 30 days (continuous), self-reported diabetes control (binary variable), PAID item on complications worry (continuous), PAID item on burnout (continuous), and current use of digital monitoring (categorical with 3 categories: participants who neither use nor intend to use monitoring tools in the future, participants who intend to use them or use them irregularly, and participants who use them regularly).

© 2021 Oikonomidi T et al. *JAMA Network Open.*

Because the minimum required effectiveness questions are rated with comparison to the patients' current monitoring, the vignette score for intrusiveness was entered in the models in comparison to the participant's current monitoring. For this, we subtracted the intrusiveness score each participant assigned to their current monitoring from the vignette intrusiveness score.

The dependent variables (minimum required effectiveness scores) were handled as ordinal. Higher values indicate higher required effectiveness.

IV. Model fit and selection of predictors

The model was fit in the imputed dataset. Correlations among independent variables were assessed for multi-collinearity. Starting from the theory-driven model specified above, we removed the weakest predictor based on its coefficient and refit the model. If this step provided improved fit (based on the Akaike information criteria [AIC], smaller values implying a better model fit), the next weakest predictor was removed, until no further improvement in AIC was achieved.

The following predictors were removed to arrive to the final model for minimum required efficacy in reducing hypoglycemic episodes: age, number of hypoglycemic episodes, current regular use of monitoring tools, 2 levels of the second vignette factor (permanently, with real-time, AI-generated treatment and lifestyle feedback, monitoring for a week before all consultations with feedback in consultation), the third vignette factor (public-sector data handling), male gender, and self-reported diabetes control (theory-driven model AIC = 6206, final model AIC = 6195).

The following predictors were removed to arrive to the final model for minimum required efficacy in preventing ophthalmologic complications: age, number of hypoglycemic episodes, current regular use of monitoring tools, 3 levels of the second vignette factor (permanently, with real-time, AI-generated treatment feedback; monitoring permanently, with real-time, AI-generated treatment and lifestyle feedback; monitoring for a week before all consultations with feedback in consultation), the third vignette factor (public-sector data handling), the PAID item on burnout, male gender, other/self-reported gender, and self-reported diabetes control (theory-driven model AIC = 5942, final model AIC = 5863).

© 2021 Oikonomidi T et al. *JAMA Network Open.*

R^2 was estimated for the models by using Nagelkerke's method.

References

1. Tanenbaum ML, Hanes SJ, Miller KM, Naranjo D, Bensen R, Hood KK. Diabetes device use in adults with type 1 diabetes: barriers to uptake and potential intervention targets. *Diabetes Care*. 2017;40(2):181-187.

2. Tanenbaum ML, Adams RN, Iturralde E, et al. From Wary Wearers to d-Embracers: Personas of Readiness to Use Diabetes Devices. *J Diabetes Sci Technol*. 2018;12(6):1101-1107. doi:10.1177/1932296818793756

3. Tran V-T, Barnes C, Montori VM, Falissard B, Ravaud P %J B medicine. Taxonomy of the burden of treatment: a multi-country web-based qualitative study of patients with chronic conditions. 2015;13(1):115.

© 2021 Oikonomidi T et al. *JAMA Network Open.*

eTable 1. Distribution of Minimum Required Effectiveness Ratings per Vignette

Vignette	n	Minimum required effectiveness for reducing hypoglycemic episodes (%)					Minimum required effectiveness for preventing ophthalmologic complications (%)				
		It could be much less effective	It could be somewhat less effective	It would have to be just as effective	It would have to be somewhat more effective	It would have to be much more effective	It could be much less effective	It could be somewhat less effective	It would have to be just as effective	It would have to be somewhat more effective	It would have to be much more effective
Glucose and PA monitoring, for a week before a specific consultation with feedback in consultation, and public-sector data handling	80	2 (2)	3 (4)	22 (28)	29 (36)	24 (30)	3 (4)	1 (1)	24 (30)	23 (29)	29 (36)
Glucose and PA monitoring, for a week before a specific consultation with feedback	78	4 (5)	1 (1)	23 (29)	21 (27)	29 (37)	3 (4)	2 (3)	23 (29)	23 (29)	27 (35)

© 2021 Oikonomidi T et al. *JAMA Network Open.*

Vignette	n	Minimum required effectiveness for reducing hypoglycemic episodes (%)					Minimum required effectiveness for preventing ophthalmologic complications (%)				
		It could be much less effective	It could be somewhat less effective	It would have to be just as effective	It would have to be somewhat more effective	It would have to be much more effective	It could be much less effective	It could be somewhat less effective	It would have to be just as effective	It would have to be somewhat more effective	It would have to be much more effective
in consultation, and private-sector data handling											
Glucose and PA monitoring, for a week before all consultations with feedback in consultation, and public-sector data handling	76	2 (3)	2 (3)	21 (28)	20 (26)	31 (41)	2 (3)	2 (3)	20 (26)	25 (33)	27 (36)
Glucose and PA monitoring, for a week before all consultations with feedback	86	2 (2)	3 (3)	28 (33)	31 (36)	22 (26)	4 (5)	0 (0)	26 (30)	30 (35)	26 (30)

© 2021 Oikonomidi T et al. *JAMA Network Open.*

Vignette	n	Minimum required effectiveness for reducing hypoglycemic episodes (%)					Minimum required effectiveness for preventing ophthalmologic complications (%)				
		It could be much less effective	It could be somewhat less effective	It would have to be just as effective	It would have to be somewhat more effective	It would have to be much more effective	It could be much less effective	It could be somewhat less effective	It would have to be just as effective	It would have to be somewhat more effective	It would have to be much more effective
in consultation, and private-sector data handling											
Glucose and PA monitoring, permanently with real-time feedback by the patient's regular physician, and public-sector data handling	79	3 (4)	4 (5)	20 (25)	28 (35)	24 (30)	2 (3)	3 (4)	24 (30)	23 (29)	27 (34)
Glucose and PA monitoring, permanently with real-time feedback by the patient's	79	1 (1)	0 (0)	19 (24)	33 (42)	26 (33)	0 (0)	1 (1)	22 (28)	32 (41)	24 (30)

© 2021 Oikonomidi T et al. *JAMA Network Open.*

Vignette	n	Minimum required effectiveness for reducing hypoglycemic episodes (%)					Minimum required effectiveness for preventing ophthalmologic complications (%)				
		It could be much less effective	It could be somewhat less effective	It would have to be just as effective	It would have to be somewhat more effective	It would have to be much more effective	It could be much less effective	It could be somewhat less effective	It would have to be just as effective	It would have to be somewhat more effective	It would have to be much more effective
regular physician, and private-sector data handling											
Glucose and PA monitoring, permanently with real-time feedback by another CP, and public-sector data handling	78	3 (4)	2 (3)	24 (31)	27 (35)	22 (28)	3 (4)	2 (3)	21 (27)	32 (41)	20 (26)
Glucose and PA monitoring, permanently with real-time feedback by another CP,	78	2 (3)	3 (4)	18 (23)	24 (31)	31 (40)	0 (0)	3 (4)	21 (27)	23 (29)	31 (40)

© 2021 Oikonomidi T et al. *JAMA Network Open.*

Vignette	n	Minimum required effectiveness for reducing hypoglycemic episodes (%)					Minimum required effectiveness for preventing ophthalmologic complications (%)				
		It could be much less effective	It could be somewhat less effective	It would have to be just as effective	It would have to be somewhat more effective	It would have to be much more effective	It could be much less effective	It could be somewhat less effective	It would have to be just as effective	It would have to be somewhat more effective	It would have to be much more effective
and private-sector data handling											
Glucose and PA monitoring, permanently with real-time, AI-generated treatment feedback, and public-sector data handling	78	2 (3)	1 (1)	21 (27)	33 (42)	21 (27)	2 (3)	2 (3)	20 (26)	32 (41)	22 (28)
Glucose and PA monitoring, permanently with real-time, AI-generated treatment	108	1 (1)	3 (3)	36 (33)	25 (23)	43 (40)	0 (0)	0 (0)	36 (33)	31 (29)	41 (38)

© 2021 Oikonomidi T et al. *JAMA Network Open.*

Vignette	n	Minimum required effectiveness for reducing hypoglycemic episodes (%)					Minimum required effectiveness for preventing ophthalmologic complications (%)				
		It could be much less effective	It could be somewhat less effective	It would have to be just as effective	It would have to be somewhat more effective	It would have to be much more effective	It could be much less effective	It could be somewhat less effective	It would have to be just as effective	It would have to be somewhat more effective	It would have to be much more effective
feedback, and private-sector data handling											
Glucose and PA monitoring, permanently with real-time, AI-generated treatment and lifestyle feedback, and public-sector data handling	80	2 (2)	2 (2)	33 (41)	24 (30)	19 (24)	1 (1)	3 (4)	30 (38)	25 (31)	21 (26)
Glucose and PA monitoring, permanently with real-time, AI-generated treatment and	90	1 (1)	2 (2)	31 (34)	22 (24)	34 (38)	0 (0)	3 (3)	32 (36)	23 (26)	32 (36)

© 2021 Oikonomidi T et al. *JAMA Network Open.*

Vignette	n	Minimum required effectiveness for reducing hypoglycemic episodes (%)					Minimum required effectiveness for preventing ophthalmologic complications (%)				
		It could be much less effective	It could be somewhat less effective	It would have to be just as effective	It would have to be somewhat more effective	It would have to be much more effective	It could be much less effective	It could be somewhat less effective	It would have to be just as effective	It would have to be somewhat more effective	It would have to be much more effective
lifestyle feedback, and private-sector data handling											
Glucose, PA and regular food monitoring, for a week before a specific consultation with feedback in consultation, and public-sector data handling	78	2 (3)	2 (3)	30 (38)	23 (29)	21 (27)	1 (1)	2 (3)	26 (33)	25 (32)	24 (31)
Glucose, PA and regular food monitoring, for a week	77	1 (1)	4 (5)	18 (23)	26 (34)	28 (36)	1 (1)	4 (5)	23 (30)	26 (34)	23 (30)

© 2021 Oikonomidi T et al. *JAMA Network Open.*

Vignette	n	Minimum required effectiveness for reducing hypoglycemic episodes (%)					Minimum required effectiveness for preventing ophthalmologic complications (%)				
		It could be much less effective	It could be somewhat less effective	It would have to be just as effective	It would have to be somewhat more effective	It would have to be much more effective	It could be much less effective	It could be somewhat less effective	It would have to be just as effective	It would have to be somewhat more effective	It would have to be much more effective
before a specific consultation with feedback in consultation, and private-sector data handling											
Glucose, PA and regular food monitoring, for a week before all consultations with feedback in consultation, and public-sector data handling	78	2 (3)	1 (1)	27 (35)	23 (29)	25 (32)	1 (1)	3 (4)	22 (28)	25 (32)	27 (35)

© 2021 Oikonomidi T et al. *JAMA Network Open.*

Vignette	n	Minimum required effectiveness for reducing hypoglycemic episodes (%)					Minimum required effectiveness for preventing ophthalmologic complications (%)				
		It could be much less effective	It could be somewhat less effective	It would have to be just as effective	It would have to be somewhat more effective	It would have to be much more effective	It could be much less effective	It could be somewhat less effective	It would have to be just as effective	It would have to be somewhat more effective	It would have to be much more effective
Glucose, PA and regular food monitoring, for a week before all consultations with feedback in consultation, and private-sector data handling	76	4 (5)	0 (0)	17 (22)	24 (32)	31 (41)	2 (3)	0 (0)	16 (21)	22 (29)	36 (47)
Glucose, PA and regular food monitoring, permanently with real-time feedback by the patient's	79	1 (1)	6 (8)	19 (24)	22 (28)	31 (39)	2 (3)	3 (4)	22 (28)	22 (28)	30 (38)

© 2021 Oikonomidi T et al. *JAMA Network Open.*

Vignette	n	Minimum required effectiveness for reducing hypoglycemic episodes (%)					Minimum required effectiveness for preventing ophthalmologic complications (%)				
		It could be much less effective	It could be somewhat less effective	It would have to be just as effective	It would have to be somewhat more effective	It would have to be much more effective	It could be much less effective	It could be somewhat less effective	It would have to be just as effective	It would have to be somewhat more effective	It would have to be much more effective
regular physician, and public-sector data handling											
Glucose, PA and regular food monitoring, permanently with real-time feedback by the patient's regular physician, and private-sector data handling	77	1 (1)	3 (4)	21 (27)	18 (23)	34 (44)	2 (3)	1 (1)	21 (27)	18 (23)	35 (45)
Glucose, PA and regular food monitoring,	77	2 (3)	3 (4)	22 (29)	17 (22)	33 (43)	0 (0)	2 (3)	22 (29)	22 (29)	31 (40)

© 2021 Oikonomidi T et al. *JAMA Network Open.*

Vignette	n	Minimum required effectiveness for reducing hypoglycemic episodes (%)					Minimum required effectiveness for preventing ophthalmologic complications (%)				
		It could be much less effective	It could be somewhat less effective	It would have to be just as effective	It would have to be somewhat more effective	It would have to be much more effective	It could be much less effective	It could be somewhat less effective	It would have to be just as effective	It would have to be somewhat more effective	It would have to be much more effective
permanently with real-time feedback by another CP, and public-sector data handling											
Glucose, PA and regular food monitoring, permanently with real-time feedback by another CP, and private-sector data handling	77	3 (4)	2 (3)	19 (25)	22 (29)	31 (40)	2 (3)	3 (4)	20 (26)	24 (31)	28 (36)

© 2021 Oikonomidi T et al. *JAMA Network Open.*

Vignette	n	Minimum required effectiveness for reducing hypoglycemic episodes (%)					Minimum required effectiveness for preventing ophthalmologic complications (%)				
		It could be much less effective	It could be somewhat less effective	It would have to be just as effective	It would have to be somewhat more effective	It would have to be much more effective	It could be much less effective	It could be somewhat less effective	It would have to be just as effective	It would have to be somewhat more effective	It would have to be much more effective
Glucose, PA and regular food monitoring, permanently with real-time, AI-generated treatment feedback, and public-sector data handling	78	1 (1)	1 (1)	19 (24)	15 (19)	42 (54)	2 (3)	2 (3)	16 (21)	24 (31)	34 (44)
Glucose, PA and regular food monitoring, permanently with real-time, AI-generated treatment	79	3 (4)	1 (1)	21 (27)	28 (35)	26 (33)	3 (4)	1 (1)	23 (29)	32 (41)	20 (25)

© 2021 Oikonomidi T et al. *JAMA Network Open.*

Vignette	n	Minimum required effectiveness for reducing hypoglycemic episodes (%)					Minimum required effectiveness for preventing ophthalmologic complications (%)				
		It could be much less effective	It could be somewhat less effective	It would have to be just as effective	It would have to be somewhat more effective	It would have to be much more effective	It could be much less effective	It could be somewhat less effective	It would have to be just as effective	It would have to be somewhat more effective	It would have to be much more effective
feedback, and private-sector data handling											
Glucose, PA and regular food monitoring, permanently with real-time, AI-generated treatment and lifestyle feedback, and public-sector data handling	78	3 (4)	4 (5)	26 (33)	23 (29)	22 (28)	3 (4)	3 (4)	22 (28)	28 (36)	22 (28)
Glucose, PA and regular food monitoring,	79	3 (4)	4 (5)	20 (25)	24 (30)	28 (35)	3 (4)	2 (3)	22 (28)	22 (28)	30 (38)

© 2021 Oikonomidi T et al. *JAMA Network Open.*

Vignette	n	Minimum required effectiveness for reducing hypoglycemic episodes (%)					Minimum required effectiveness for preventing ophthalmologic complications (%)				
		It could be much less effective	It could be somewhat less effective	It would have to be just as effective	It would have to be somewhat more effective	It would have to be much more effective	It could be much less effective	It could be somewhat less effective	It would have to be just as effective	It would have to be somewhat more effective	It would have to be much more effective
permanently with real-time, AI-generated treatment and lifestyle feedback, and private-sector data handling											
Glucose, PA and occasional food monitoring, for a week before a specific consultation with feedback in consultation, and public-sector data handling	80	1 (1)	6 (8)	20 (25)	29 (36)	24 (30)	1 (1)	6 (8)	18 (22)	34 (42)	21 (26)

© 2021 Oikonomidi T et al. *JAMA Network Open.*

Vignette	n	Minimum required effectiveness for reducing hypoglycemic episodes (%)					Minimum required effectiveness for preventing ophthalmologic complications (%)				
		It could be much less effective	It could be somewhat less effective	It would have to be just as effective	It would have to be somewhat more effective	It would have to be much more effective	It could be much less effective	It could be somewhat less effective	It would have to be just as effective	It would have to be somewhat more effective	It would have to be much more effective
Glucose, PA and occasional food monitoring, for a week before a specific consultation with feedback in consultation, and private-sector data handling	78	1 (1)	6 (8)	16 (21)	28 (36)	27 (35)	2 (3)	5 (6)	18 (23)	21 (27)	32 (41)
Glucose, PA and occasional food monitoring, for a week before all consultations with feedback in consultation,	77	2 (3)	3 (4)	18 (23)	22 (29)	32 (42)	4 (5)	2 (3)	15 (19)	24 (31)	32 (42)

© 2021 Oikonomidi T et al. *JAMA Network Open.*

Vignette	n	Minimum required effectiveness for reducing hypoglycemic episodes (%)					Minimum required effectiveness for preventing ophthalmologic complications (%)				
		It could be much less effective	It could be somewhat less effective	It would have to be just as effective	It would have to be somewhat more effective	It would have to be much more effective	It could be much less effective	It could be somewhat less effective	It would have to be just as effective	It would have to be somewhat more effective	It would have to be much more effective
and public-sector data handling											
Glucose, PA and occasional food monitoring, for a week before all consultations with feedback in consultation, and private-sector data handling	77	1 (1)	2 (3)	23 (30)	22 (29)	29 (38)	1 (1)	0 (0)	19 (25)	28 (36)	29 (38)
Glucose, PA and occasional food monitoring,	79	1 (1)	1 (1)	18 (23)	21 (27)	38 (48)	0 (0)	1 (1)	14 (18)	29 (37)	35 (44)

© 2021 Oikonomidi T et al. *JAMA Network Open.*

Vignette	n	Minimum required effectiveness for reducing hypoglycemic episodes (%)					Minimum required effectiveness for preventing ophthalmologic complications (%)				
		It could be much less effective	It could be somewhat less effective	It would have to be just as effective	It would have to be somewhat more effective	It would have to be much more effective	It could be much less effective	It could be somewhat less effective	It would have to be just as effective	It would have to be somewhat more effective	It would have to be much more effective
permanently with real-time feedback by their regular physician, and public-sector data handling											
Glucose, PA and occasional food monitoring, permanently with real-time feedback by their regular physician, and private-sector data handling	79	1 (1)	6 (8)	20 (25)	21 (27)	31 (39)	1 (1)	5 (6)	24 (30)	23 (29)	26 (33)

© 2021 Oikonomidi T et al. *JAMA Network Open.*

Vignette	n	Minimum required effectiveness for reducing hypoglycemic episodes (%)					Minimum required effectiveness for preventing ophthalmologic complications (%)				
		It could be much less effective	It could be somewhat less effective	It would have to be just as effective	It would have to be somewhat more effective	It would have to be much more effective	It could be much less effective	It could be somewhat less effective	It would have to be just as effective	It would have to be somewhat more effective	It would have to be much more effective
Glucose, PA and occasional food monitoring, permanently with real-time feedback by another CP, and public-sector data handling	77	2 (3)	5 (6)	14 (18)	28 (36)	28 (36)	1 (1)	3 (4)	12 (16)	35 (45)	26 (34)
Glucose, PA and occasional food monitoring, permanently with real-time feedback by another CP,	77	4 (5)	0 (0)	21 (27)	20 (26)	32 (42)	3 (4)	1 (1)	18 (23)	24 (31)	31 (40)

© 2021 Oikonomidi T et al. *JAMA Network Open.*

Vignette	n	Minimum required effectiveness for reducing hypoglycemic episodes (%)					Minimum required effectiveness for preventing ophthalmologic complications (%)				
		It could be much less effective	It could be somewhat less effective	It would have to be just as effective	It would have to be somewhat more effective	It would have to be much more effective	It could be much less effective	It could be somewhat less effective	It would have to be just as effective	It would have to be somewhat more effective	It would have to be much more effective
and private-sector data handling											
Glucose, PA and occasional food monitoring, permanently with real-time, AI-generated treatment feedback, and public-sector data handling	78	3 (4)	5 (6)	23 (29)	26 (33)	21 (27)	2 (3)	3 (4)	28 (36)	20 (26)	25 (32)
Glucose, PA and occasional food monitoring,	79	2 (3)	2 (3)	18 (23)	24 (30)	33 (42)	2 (3)	3 (4)	15 (19)	24 (30)	35 (44)

© 2021 Oikonomidi T et al. *JAMA Network Open.*

Vignette	n	Minimum required effectiveness for reducing hypoglycemic episodes (%)					Minimum required effectiveness for preventing ophthalmologic complications (%)				
		It could be much less effective	It could be somewhat less effective	It would have to be just as effective	It would have to be somewhat more effective	It would have to be much more effective	It could be much less effective	It could be somewhat less effective	It would have to be just as effective	It would have to be somewhat more effective	It would have to be much more effective
permanently with real-time, AI-generated treatment feedback, and private-sector data handling											
Glucose, PA and occasional food monitoring, permanently with real-time, AI-generated treatment and lifestyle feedback, and public-sector data handling	79	2 (3)	1 (1)	14 (18)	30 (38)	32 (41)	2 (3)	4 (5)	18 (23)	27 (34)	28 (35)

© 2021 Oikonomidi T et al. *JAMA Network Open.*

Vignette	n	Minimum required effectiveness for reducing hypoglycemic episodes (%)					Minimum required effectiveness for preventing ophthalmologic complications (%)				
		It could be much less effective	It could be somewhat less effective	It would have to be just as effective	It would have to be somewhat more effective	It would have to be much more effective	It could be much less effective	It could be somewhat less effective	It would have to be just as effective	It would have to be somewhat more effective	It would have to be much more effective
Glucose, PA and occasional food monitoring, permanently with real-time, AI-generated treatment and lifestyle feedback, and private-sector data handling	77	5 (6)	5 (6)	24 (31)	23 (30)	20 (26)	4 (5)	6 (8)	20 (26)	21 (27)	26 (34)

PA, physical activity; CP, care professional; AI, artificial intelligence

© 2021 Oikonomidi T et al. *JAMA Network Open.*

eTable 2. Cumulative Link Mixed Model in the Complete-Case Data Set

	Minimum required effectiveness					
	Reducing hypoglycemic episodes [a]			Preventing ophthalmologic complications [b]		
**Predictors**	**Estimate (SE)**	**OR (95% CI)**	**P**	**Estimate (SE)**	**OR (95% CI)**	**P**
Intercept: Much less | Somewhat less	-5.5 (0.47)	0.00 (0.00–0.01)	<0.001	-7.6 (0.57)	0.00 (0.00–0.00)	<0.001
Intercept: Somewhat less | Just as	-3.91 (0.44)	0.02 (0.01–0.05)	<0.001	-5.39 (0.51)	0.00 (0.00–0.01)	<0.001
Intercept: Just as | Somewhat more	0.19 (0.42)	1.20 (0.53–2.73)	0.66	-0.61 (0.47)	0.54 (0.21–1.38)	0.20
Intercept: Somewhat more | Much more	3.01 (0.43)	20.32 (8.83–46.75)	<0.001	2.75 (0.48)	15.65 (6.12–40.04)	<0.001
**Vignette-level predictors**						

© 2021 Oikonomidi T et al. *JAMA Network Open.*

Monitoring tools (reference category: glucose and PA)						
Glucose, PA and regular food monitoring	0.39 (0.13)	1.48 (1.14–1.92)	0.003	0.3 (0.14)	1.35 (1.03–1.77)	0.03
Glucose, PA and occasional food monitoring	0.47 (0.13)	1.60 (1.24–2.08)	<0.001	0.35 (0.14)	1.42 (1.08–1.87)	0.01
Duration/feedback loop (ref. cat.: For a week before a specific consultation, with feedback in consultation)						

© 2021 Oikonomidi T et al. *JAMA Network Open.*

Permanently, with real-time feedback by the patient's regular physician	0.26 (0.16)	1.30 (0.95–1.77)	0.10	0.21 (0.15)	1.23 (0.91–1.67)	0.17
Permanently, with real-time feedback by another care professional	0.41 (0.16)	1.51 (1.09–2.07)	0.01	0.47 (0.16)	1.60 (1.18–2.18)	0.003
Permanently, with real-time, artificial intelligence-generated treatment feedback [c]	0.38 (0.16)	1.46 (1.07–1.98)	0.02			

© 2021 Oikonomidi T et al. *JAMA Network Open.*

Permanently, with real-time, artificial intelligence-generated treatment and lifestyle feedback [c]	0.14 (0.16)	1.15 (0.84–1.56)	0.39			
Intrusiveness rating	0.38 (0.06)	1.47 (1.30–1.65)	<0.001	0.44 (0.06)	1.55 (1.37–1.76)	<0.001
**Participant characteristics**						
Intends to use monitoring tools for health or wellbeing purposes, or uses	-0.26 (0.33)	0.77 (0.40–1.47)	0.43	-0.29 (0.38)	0.75 (0.36–1.57)	0.44

© 2021 Oikonomidi T et al. *JAMA Network Open.*

them irregularly (reference category: does not use them and does not intend to)						
Feeling "burned out" by the constant effort needed to manage diabetes (PAID questionnaire item) [c]	0.15 (0.13)	1.16 (0.90–1.50)	0.25			
Worrying about the future and the possibility of serious	0.23 (0.14)	1.26 (0.96–1.66	0.09	0.47 (0.13)	1.60 (1.25–2.05)	<0.001

© 2021 Oikonomidi T et al. *JAMA Network Open.*

complications (PAID questionnaire item)						
Insulin use (ref. cat.: no insulin use)						
Insulin shots	0.72 (0.31)	2.05 (1.12–3.76)	0.02	0.39 (0.35)	1.48 (0.74–2.96)	0.27
Insulin pump	1.09 (0.33)	2.99 (1.57–5.68)	0.001	0.69 (0.37)	1.99 (0.96–4.09)	0.06
Country of residence (ref. cat.: France)						
Countries other than France, United States and Canada	-0.72 (0.31)	0.49 (0.27–0.89)	0.02	-1.33 (0.36)	0.27 (0.13–0.53)	<0.001
United States	-0.3 (0.39)	0.74 (0.34–1.59)	0.44	-1.18 (0.45)	0.31 (0.13–0.74)	0.008

© 2021 Oikonomidi T et al. *JAMA Network Open.*

Canada	-0.07 (0.34)	0.94 (0.48–1.82)	0.85	-0.36 (0.39)	0.70 (0.32–1.49)	0.35
Gender: Prefers to self-describe (ref. cat.: woman)[c]	0.51 (0.63)	1.67 (0.48–5.72)	0.42			

SE, standard error; OR, odds ratio; 95% CI, 95% confidence interval

[a] AIC = 5188, pseudo-R2 = 0.06 (estimated for the model vs the null using Nagelkerke's method).

[b] AIC = 4861, pseudo-R2 = 0.07 (estimated for the model vs the null using Nagelkerke's method).

[c] This variable was not included in the final model for minimum required effectiveness in preventing ophthalmologic complications

© 2021 Oikonomidi T et al. *JAMA Network Open.*

eFigure 1. Study Flowchart

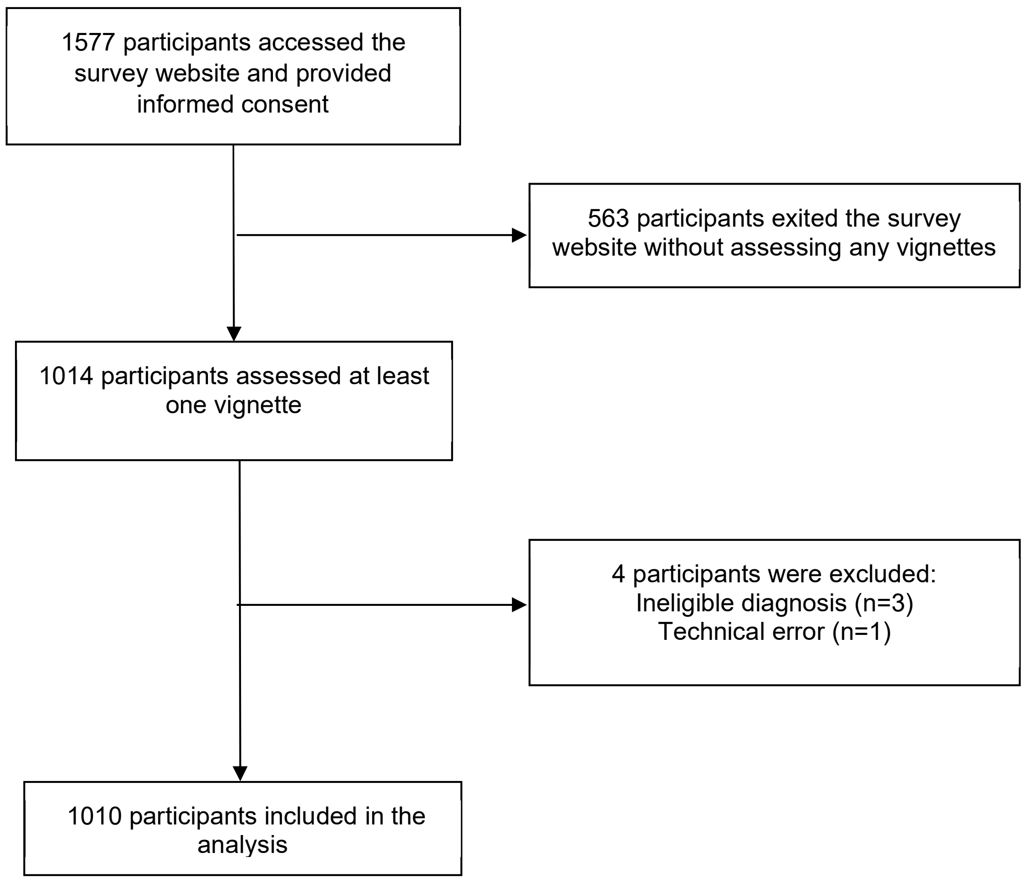

© 2021 Oikonomidi T et al. *JAMA Network Open.*

eFigure 2. Subgroup Analysis by Insulin Use Subgroup for Reducing Hypoglycemic Episodes

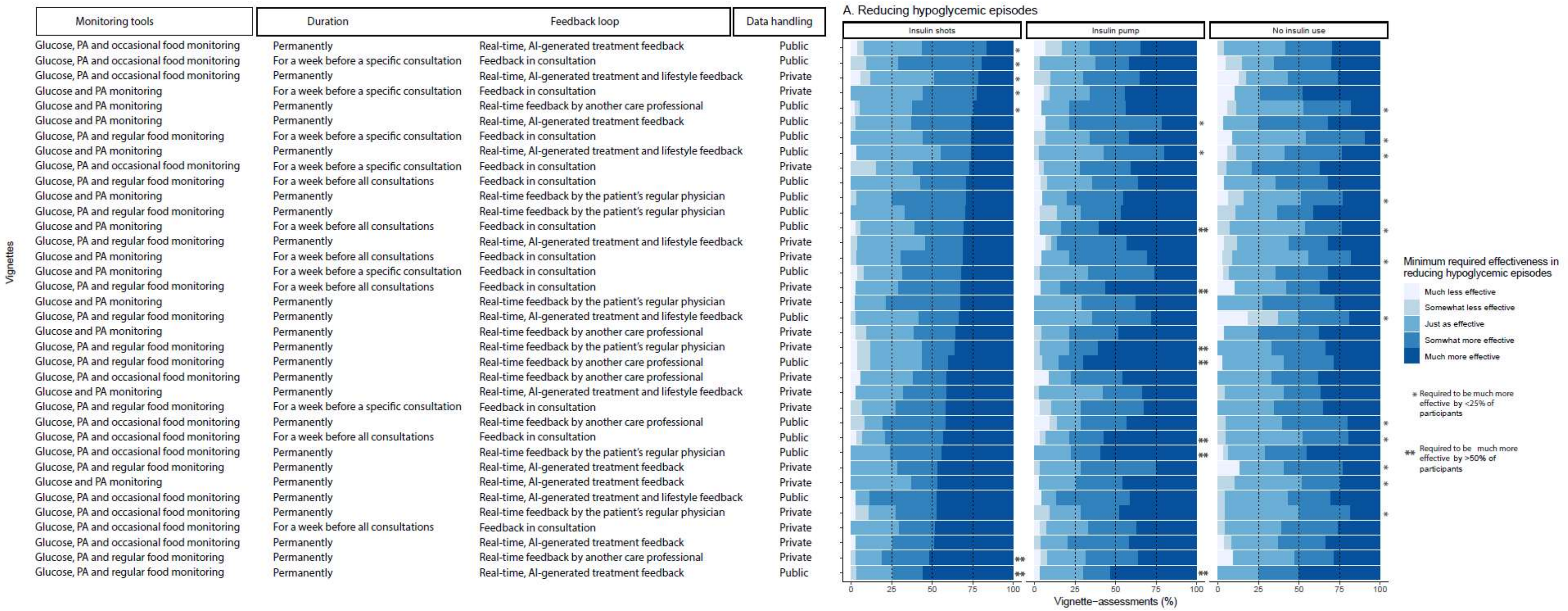

The figure shows the minimum required effectiveness of remote digital monitoring (RDM) in reducing hypoglycemic episodes by insulin use subgroup. The vignettes are ranked by the proportion of vignette assessments requiring that the vignette RDM be much more effective than the participant's current monitoring for the subgroup of insulin shots. The asterisks show vignettes with high and low minimum required effectiveness. There were 5 vignettes with low minimum required effectiveness for participants who use insulin shots, 2 for participants who use an insulin pump and 12 for those who use no insulin. There were 2 vignettes with high minimum required effectiveness for participants who use insulin shots, 7 for participants who use an insulin pump and none for those who use no insulin.

© 2021 Oikonomidi T et al. *JAMA Network Open.*

eFigure 3. Subgroup Analysis by Insulin Use Subgroup for Preventing Ophthalmologic Complications

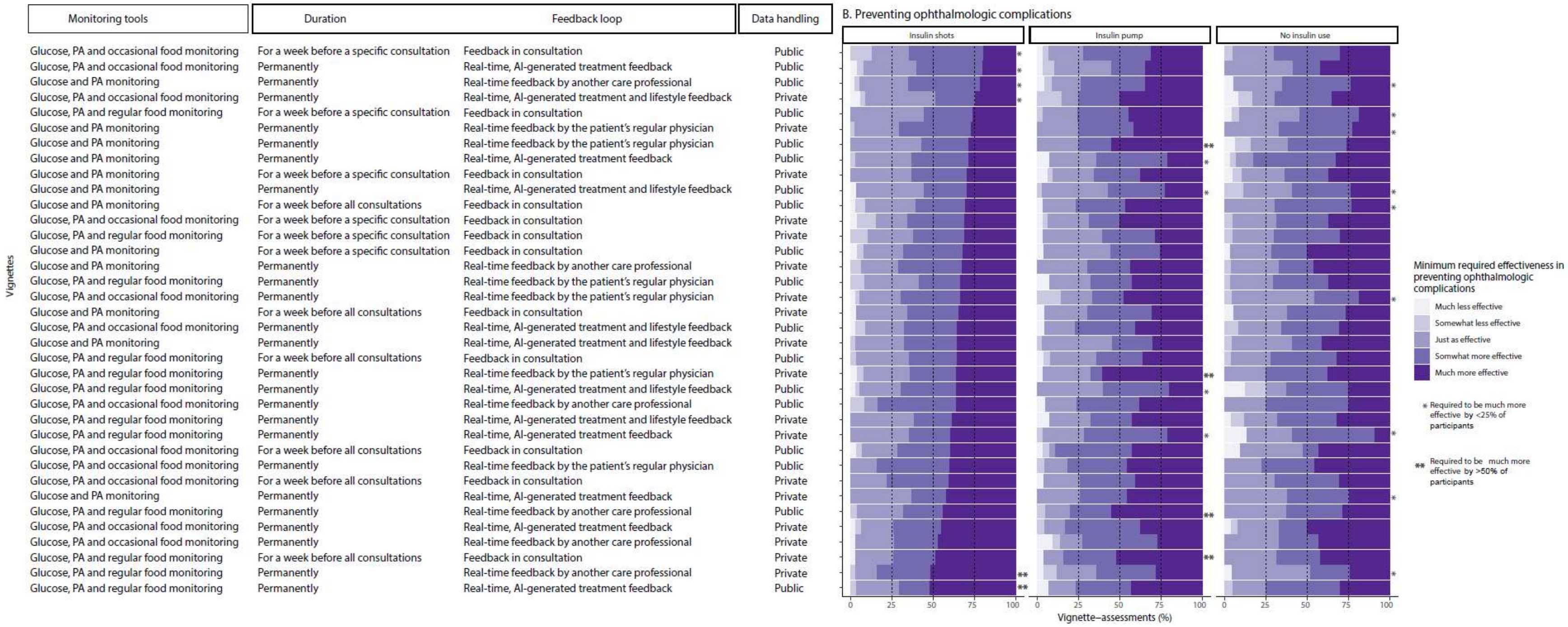

The figure shows the minimum required effectiveness of remote digital monitoring (RDM) in preventing ophthalmologic complications by insulin use subgroup. The vignettes are ranked by the proportion of vignette assessments requiring that the vignette RDM be much more effective than the participant's current monitoring for the insulin shots subgroup. The asterisks show vignettes with high and low minimum required effectiveness. There were 4 vignettes with low minimum required effectiveness for participants who use insulin shots, 4 for participants who use an insulin pump and 9 for those who use no insulin. There were 2 vignettes with high minimum required effectiveness for participants who use insulin shots, 4 for participants who use an insulin pump and none for those who use no insulin.

© 2021 Oikonomidi T et al. *JAMA Network Open.*

eFigure 4. Subgroup Analysis by Diabetes Type Subgroup for Reducing Hypoglycemic Episodes

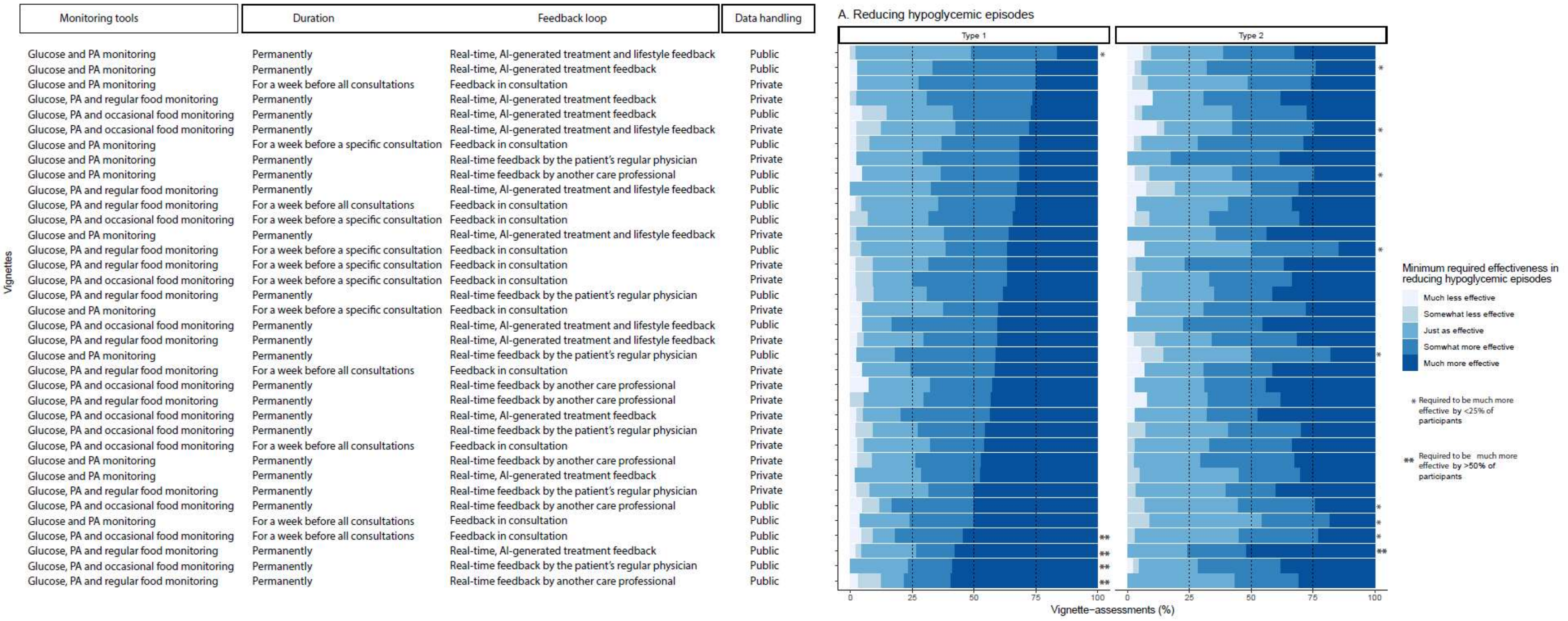

The figure shows the minimum required effectiveness of remote digital monitoring (RDM) in reducing hypoglycemic episodes by diabetes type subgroup. The vignettes are ranked by the proportion of vignette assessments requiring that the vignette RDM be much more effective than the participant's current monitoring for the subgroup of participants with type 1 diabetes. The asterisks show vignettes with high and low minimum required effectiveness. There was 1 vignette with low minimum required effectiveness for participants who had type 1 diabetes, and 8 for participants who had type 2 diabetes. There were 4 vignettes with high minimum required effectiveness for participants who had type 1 diabetes, and 1 for participants who had type 2 diabetes.

© 2021 Oikonomidi T et al. *JAMA Network Open.*

eFigure 5. Subgroup Analysis by Diabetes Type Subgroup for Preventing Ophthalmologic Complications

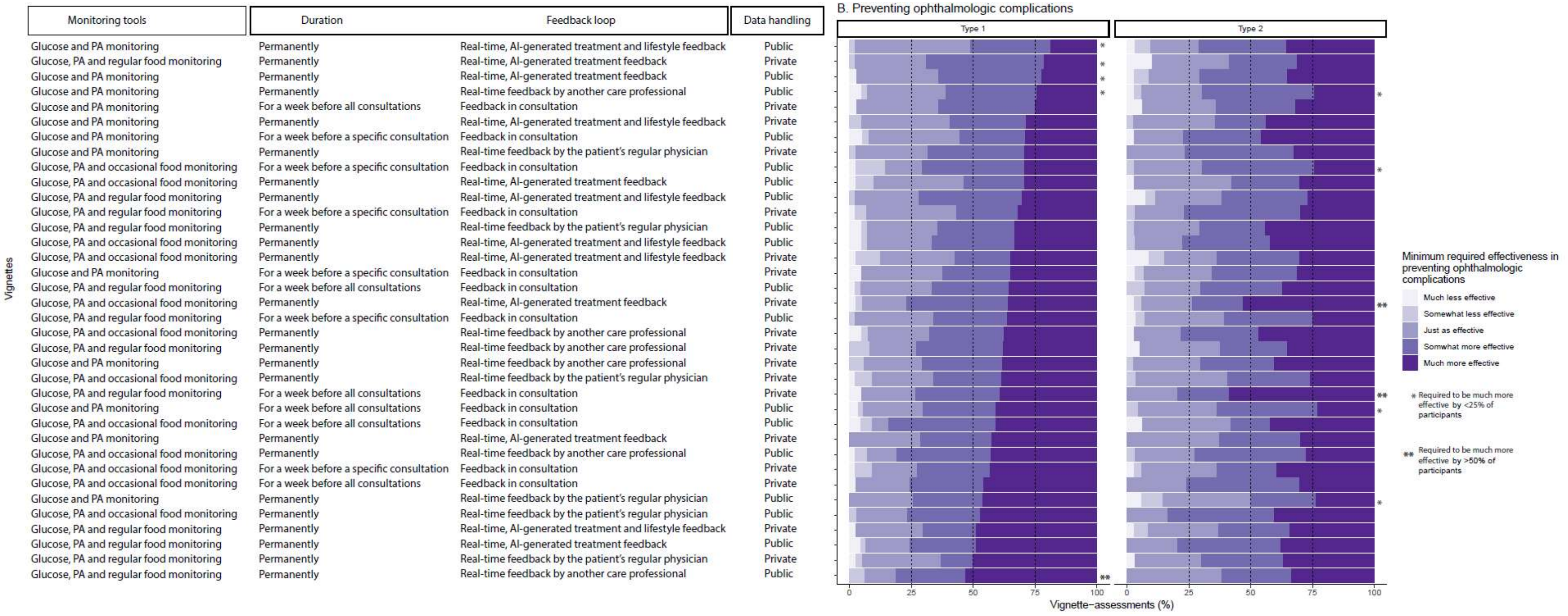

The figure shows the minimum required effectiveness of remote digital monitoring (RDM) by diabetes type subgroup, for preventing ophthalmologic complications. The vignettes are ranked by the proportion of vignette assessments requiring that the vignette RDM be much more effective than the participant's current monitoring for the subgroup of participants with type 1 diabetes. The asterisks show vignettes with low and high minimum required effectiveness. There are more outlier vignettes with low minimum required effectiveness in the type 2 subgroup. There were 4 vignettes with low minimum required effectiveness for both participants with type 1 and type 2 diabetes (see Supplemental Figure 7 for vignette description). There was 1 vignette with high minimum required effectiveness for participants who had type 1 diabetes, and 2 for participants who had type 2 diabetes.

© 2021 Oikonomidi T et al. *JAMA Network Open.*
